# Albumin in early septic shock resuscitation: examination of plasma and urine inflammatory markers

**DOI:** 10.1186/cc10861

**Published:** 2012-03-20

**Authors:** A Fox-Robichaud, C Leger, KD Burns, E Sabri, B Lo, P Kubes, LA McIntyre

**Affiliations:** 1 McMaster University, Hamilton, Canada; 2 University of Calgary, Canada; 3 Ottawa Hospital Research Institute, Ottawa, Canada

## Introduction

A recent meta-analysis has suggested that albumin may be beneficial in sepsis; however, there is no clear biological rationale for the pharmacological use of this negative acute-phase protein. Our objective was to describe the temporal production of plasma and urine biomarkers in a pilot study of early septic shock patients resuscitated with either 5% albumin or normal saline.

## Methods

Patients presenting in early septic shock received albumin or saline in a randomized, double-blind pilot study. Blood and urine was collected at enrolment and 6, 12, 24, 72 hours and 7 days later and processed using standard operating procedures. A panel of 27 cytokines, chemokines and growth factors was measured by multiplex technology. Mean values were separated by treatment and analyzed using R to generate heat maps, by principal component analysis (PCA) and hierarchal clustering. Urinary neutrophil gelatinase-associated lipocalin (NGAL) was measured by ELISA.

## Results

Twenty-five patients (median age 66 years, median APACHE II score 26) received albumin (median amount 3 l) and 21 (median age 62 years, median APACHE II score 22) received normal saline (median amount 3.5 l) as study fluid over 7 days. PCA revealed that 60% of the variance in the chemokines was accounted for with the first two components. Analyzing the first component using a threshold of greater than 0.5 or -0.5 we saw a clustering of IL-17, IL-12p70, IL-9 and IL-5. Heat map analysis suggests that by 72 hours albumin-resuscitated patients are distinguished by the cluster of IL-17, IL-9 and Il-12p70 and VEGF when compared to saline. Hierarchal clustering also separates IL-17, IL-19, IL-12p70 and IL-2 in the albumin-treated patients but not the saline-treated patients at 72 hours. At enrolment, mean urine NGAL levels were greater than 1,000 ng/ml (albumin 1,121 ± 2,172 (*n *= 21), saline 1,375 ± 3,197 (*n *= 17)). Over the next 24 hours there was a marked increase in urine NGAL in the saline-resuscitated patients, peaking at 5,793 ± 15,948 ng/ml, whereas levels remain blunted over the first 12 hours, peaking at 2,216 ± 3,177 ng/ml at 24 hours in the albumin group.

## Conclusion

In this cohort of patients treated with albumin or saline in early septic shock, there appeared to be a marked increase in the clustering of early T-cell-mediated immune responses. Also striking was the blunted rise in urine NGAL over time for patients in the albumin fluid group. These results should be considered hypothesis generating and prompt further studies to explore possible biological mechanisms for albumin resuscitation in sepsis.

